# An Integrative Approach to Investigate the Mode of Action of (−)-Dendroparishiol in Bacterial Meningitis: Computer-Aided Estimation of Biological Activity and Network Pharmacology

**DOI:** 10.3390/ijms24098072

**Published:** 2023-04-29

**Authors:** Thanchanok Limcharoen, Peththa Wadu Dasuni Wasana, Hasriadi, Pornpoom Angsuwattana, Chawanphat Muangnoi, Sakan Warinhomhoun, Tassanee Ongtanasup, Boonchoo Sritularak, Opa Vajragupta, Pornchai Rojsitthisak, Pasarapa Towiwat

**Affiliations:** 1 Department of Applied Thai Traditional Medicine, School of Medicine, Walailak University, Nakhon Si Thammarat 80160, Thailand; 2 Research Center in Tropical Pathobiology, Walailak University, Nakhon Si Thammarat 80160, Thailand; 3 Department of Pharmacy, Faculty of Allied Health Sciences, University of Ruhuna, Galle 80000, Sri Lanka; 4 Department of Pharmacology and Physiology, Faculty of Pharmaceutical Sciences, Chulalongkorn University, Bangkok 10330, Thailand; 5 Department of Food and Pharmaceutical Chemistry, Faculty of Pharmaceutical Sciences, Chulalongkorn University, Bangkok 10330, Thailand; 6 Institute of Nutrition, Mahidol University, Salaya, Nakhon Pathom 73170, Thailand; 7 Department of Pharmacognosy and Pharmaceutical Botany, Faculty of Pharmaceutical Sciences, Chulalongkorn University, Bangkok 103300, Thailand; 8 Molecular Probes for Imaging Research Network, Faculty of Pharmaceutical Sciences, Chulalongkorn University, Bangkok 103300, Thailand; 9 Center of Excellence in Natural Products for Ageing and Chronic Diseases, Chulalongkorn University, Bangkok 10330, Thailand

**Keywords:** *Dendrobium parishii*, anti-neuroinflammatory effects, microglia, network pharmacology

## Abstract

Bacterial meningitis remains one of the most prevalent infectious diseases worldwide. Although advances in medical care have improved mortality and morbidity, neurological complications remain high. Therefore, aside from antibiotics, therapeutic adjuvants targeting neuroinflammation are essential to combat the long-term neuronal sequelae of bacterial meningitis. In the present study, we propose (−)-dendroparishiol as a potential add-on therapy to improve neuroinflammation associated with bacterial meningitis. The biological activity of (−)-dendroparishiol was first predicted by computational analysis and further confirmed in vitro using a cell-based assay with LPS-induced BV-2 microglial cells. Biological pathways involved with (−)-dendroparishiol were identified by applying network pharmacology. Computational predictions of biological activity indicated possible attenuation of several inflammatory processes by (−)-dendroparishiol. In LPS-induced BV-2 microglial cells, (−)-dendroparishiol significantly reduced the expression of inflammatory mediators: iNOS, NO, COX-2, IL-6, and TNF-α. Molecular docking results demonstrated the potential iNOS and COX-2 inhibitory activity of (−)-dendroparishiol. Network pharmacological analysis indicated the plausible role of (−)-dendroparishiol in biological processes involved in oxidative stress and neuroinflammation with enrichment in neuroinflammatory pathways. Overall, this study provides scientific evidence for the potential application of (−)-dendroparishiol in the management of bacterial meningitis-associated neuroinflammation.

## 1. Introduction

Bacterial meningitis is a disease that arises as a result of inflammation of the meninges surrounding the brain and spinal cord and remains one of the most prevalent infectious diseases worldwide [1]. Blood-borne bacteria that invade the brain parenchyma are primarily responsible for causing such inflammation. The most common etiological agents of bacterial meningitis are *Streptococcus pneumoniae*, *Neisseria meningitidis*, and *Haemophilus influenzae* [2]. Following their passage across the blood–brain barrier, bacteria interact with the fundamental units of the central nervous system, including neurons and glial cells. Although there is an ongoing investigation into the specific molecular mechanism by which assorted pathogens interact with neurons, it is clear that there is a relationship between bacterial interaction with neurons and neuroinflammatory responses within the brain that can lead to the death of neurons [3]. Additionally, a number of clinical studies have indicated that meningitis can cause dementia and other neurodegenerative diseases [2]. In addition, up to 50% of survivors of bacterial meningitis often suffer from severe neurological complications after surviving the disease [4]. Thus, healthcare systems are greatly affected by the costs associated with post-meningitis sequelae [5]. Antibiotics commonly used for bacterial meningitis include penicillin-type antibiotics and third-generation cephalosporins [6,7]. In addition to antibiotics, anti-inflammatory agents are used as adjunctive therapy for bacterial meningitis, often combined with antibiotics [8]. Dexamethasone has been shown to be effective in combating bacterial meningitis by suppressing inflammatory responses caused by bacteria. However, the use of dexamethasone can also have detrimental effects on hippocampal function, as shown by increased neuronal apoptosis [9]. It is, therefore, crucial for the discovery of new therapeutic agents that can lower inflammatory response associated with bacterial meningitis with minor side effects.

*Dendrobium* species, one of the largest genera in the *Orchidaceae* family, have been used in traditional Chinese medicine due to their multiple pharmacological properties [10,11]. Several *Dendrobium* species have been found to possess anti-inflammatory activity, including *Dendrobium nobile* [12], *Dendrobium huoshanense* [13], and *Dendrobium moniliforme* [14]. Specifically, recent research suggests *Dendrobium moniliforme* is a potential remedial agent for inflammatory diseases mediated by Gram-positive bacteria, including meningitis [14]. These findings suggest that bioactive compounds derived from *Dendrobium* species could serve as potential novel therapeutic leads against bacterial meningitis by suppressing inflammatory mediators. Among several medicinal plants of *Dendrobium* spp., *Dendrobium parishii*, known as “Ueang Khrang Sai San” in Thailand, has been reported to possess both anti-inflammatory and antioxidant properties [11,15]. (−)-Dendroparishiol is a novel bibenzyl-dihydrophenanthrene derivative isolated from *Dendrobium parishii* whole plant with potent antioxidant and anti-inflammatory properties. The antioxidant effect is mediated by increasing the levels of superoxide dismutase (SOD), glutathione peroxidase (GPx), and catalase (CAT), and the anti-inflammatory effect is exerted via decreasing the expression of inducible nitric oxide synthase (iNOS) and cyclooxygenase-2 (COX-2) [15]. Thus, (−)-dendroparishiol could be a potential lead compound in the treatment of bacterial meningitis. However, the exact mechanism of (−)-dendroparishiol in bacterial meningitis remains unknown.

Computational approaches are widely used and have gained recent attention in drug discovery. This approach enables pharmaceutical companies and academia to work effectively and reduce costs, time, and resources during the drug discovery process [16]. The details of disease targets, as well as lead compounds, are readily available in numerous databases. With computational analysis, the potential biological activity of lead compounds and their biological targets can be elucidated [16,17]. With information on active compounds, their biological activity can be predicted by the structure-activity relationships [18]. The concept of this approach is that compounds with similar structures will possess at least similar biological activity. As such, databases of compounds with their biological activities are used as a reference to predict the activity of the compound of interest. In addition, the network-based analysis approach, including network pharmacology, has also been introduced as a platform to investigate the role of active compounds through systems biology. In this approach, several databases and libraries are used to retrieve active compounds and disease targets, which are then analyzed for their biological effects and pathways [19]. Network pharmacology has emerged as a powerful tool to explore the complex interactions between bioactive compounds and their target proteins, particularly in the context of traditional medicine [20,21]. This holistic approach facilitates the identification of multiple targets and pathways involved in the therapeutic effects of a compound, thereby providing a comprehensive understanding of its mode of action.

**Figure 1:**
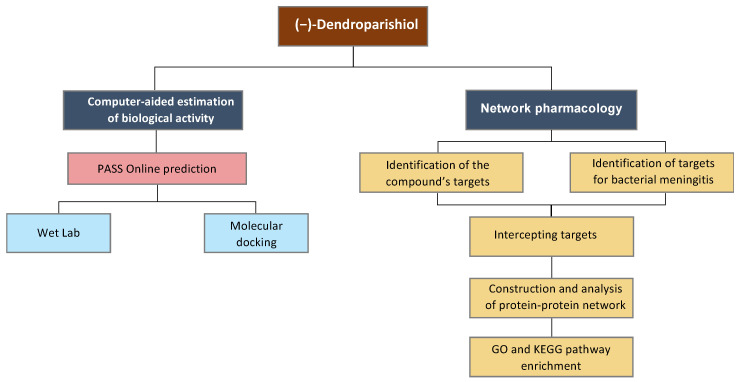
Schematic presentation of the experimental design.

Thus, in the present study, the potential targets of (−)-dendroparishiol in bacterial meningitis were identified using computational-based analysis, and the targets were verified using an in vitro cell-based model of neuroinflammation and molecular docking. Further, the detailed mechanisms of (−)-dendroparishiol in bacterial meningitis were elucidated through the network pharmacology approach (Figure 1). The findings of this study provide preliminary evidence for the use of (−)-dendroparishiol in the management of neuroinflammation associated with bacterial meningitis.

## 2. Results and Discussion

### 2.1. Profiles of Estimated Biological Activity of (−)-Dendroparishiol

**Figure 2:**
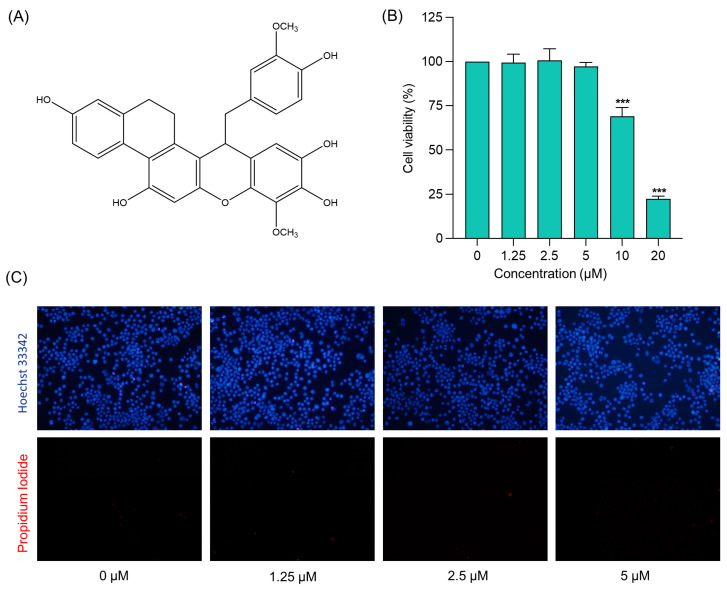
Effects of (−)-dendroparishiol on the viability of BV-2 microglial cells. (**A**) Structure of (−)-dendroparishiol (Group: Bibenzyl-dihydro-phenanthrenes, MW 514). (**B**) Effect of (−)-dendroparishiol on BV-2 cell viability assessed by MTT assay. (**C**) Effect of (−)-dendroparishiol at 1.25–5 µM on apoptosis and necrosis visualized by Hoechst 33342/PI staining. Data show the mean ± SD values of three independent experiments. *** *p* < 0.001 compared with the control group, ANOVA followed by Dunnett’s post hoc test.

Neuroinflammation associated with bacterial meningitis is linked with the increased expression of proinflammatory mediators. Therefore, targeting these mediators could contribute to attenuating meningitis progression [2]. In the present study, the biological activity of (−)-dendroparishiol (Figure 2A) was first predicted using the PASS online database (http://www.way2drug.com/passonline/, accessed on 6 January 2023). The category was adjusted to Pa (pharmacologically active) > Pi (pharmacologically inactive). As per the results, (−)-dendroparishiol potentially exhibits a wide range of biological activities, including anti-inflammatory activity (Table 1). The biological activities of (−)-dendroparishiol associated with the inhibition of inflammatory pathways were further confirmed in vitro. Most of the predicted biological activities inhibited by (−)-dendroparishiol are associated with the pathophysiology of bacterial meningitis [22]. During bacterial infections, the resident cells in the perivascular space and meninges release proinflammatory mediators in response to propagating bacteria and their components: lipopolysaccharides, lipoteichoic and teichoic acid, and peptidoglycans. Early in the course of bacterial infection, tumor necrosis factor (TNF), interleukin-1 (IL-1), and interleukin-6 (IL-6) are released, triggering a cascade of additional proinflammatory mediators such as cytokines, chemokines, prostaglandins, matrix metalloproteinases (MMPs), nitric oxide (NO) and reactive oxygen species (ROS) [3]. Thus, the potential attenuation of most of these inflammatory responses by (−)-dendroparishiol indicates its potential use in the management of bacterial meningitis.

### 2.2. Cytotoxicity Profile of (−)-Dendroparishiol in BV-2 Microglial Cells

The cytotoxicity profile of (−)-dendroparishiol on the viability of BV-2 cells was evaluated by the MTT assay. The results indicated that concentrations of (−)-dendroparishiol up to 5 µM did not significantly affect cell viability in BV-2 cells (Figure 2B). Moreover, Hoechst 33342/PI staining was used to validate the cytotoxicity results obtained. As shown in Figure 2C, cells treated with (−)-dendroparishiol 1.25–5 µM showed no significant induction of cellular apoptosis or necrosis. In subsequent experiments, (5 μM)-dendroparishiol was used to establish sustained and effective activity, enabling investigation of its anti-inflammatory effects without the confounding factors of cell death.

### 2.3. Effects of (−)-Dendroparishiol on Pro-Inflammatory Mediators in LPS-Stimulated BV-2 Cells

**Figure 3:**
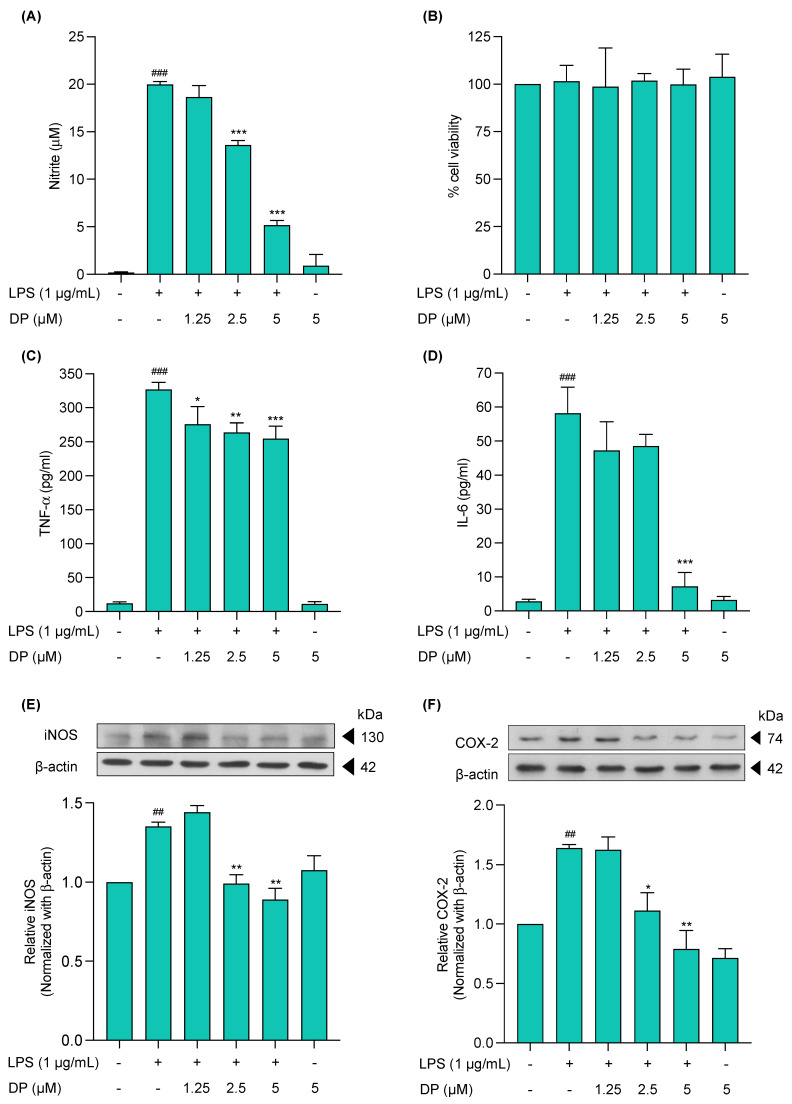
Anti-inflammatory effects of (−)-dendroparishiol on LPS-induced BV-2 microglial cells. (**A**) Effect of (−)-dendroparishiol on nitric oxide release. (**B**) Cytotoxicity of (−)-dendroparishiol on LPS-induced BV-2 cells. Effects of (−)-dendroparishiol on proinflammatory cytokine expression in LPS-stimulated BV-2 cells: (**C**) TNF-α and (**D**) IL-6 levels. Effects of (−)-dendroparishiol on protein expressions of (**E**) iNOS and (**F**) COX-2. The values of iNOS and COX-2 proteins were normalized with β-actin. Data show the mean ± SD values of three independent experiments. ^##^ *p* < 0.01 and ^###^ *p* < 0.001 compared to control group, * *p* < 0.05, ** *p* < 0.01, and *** *p* < 0.001 compared to LPS-control group, ANOVA followed by Tukey post hoc test. DP, (−)-dendroparishiol.

In line with the results of computer-based activity prediction data, the anti-inflammatory effect of (−)-dendroparishiol was initially screened in LPS-induced BV-2 cells by measuring NO expression in the culture media. Briefly, the cells were pretreated with (−)-dendroparishiol for 1 h, followed by induction with LPS (1 μg/mL) for 24 h. The NO expression in the cell culture media was then measured using the Griess reaction. The results demonstrated that LPS-treated BV-2 cells significantly increased the nitrite levels (19.99 ± 0.28 µM) compared to the control group (0.19 ± 0.09). Treatment of cells with (−)-dendroparishiol led to a concentration-dependent decrease in LPS-induced NO production. (−)-Dendroparishiol at concentrations of 2.5 and 5 µM significantly lowered the levels of nitrite by 32.1% (13.61 ± 0.45 µM) and 74.5% (5.16 ± 0.47 µM), respectively, compared to the LPS only group (Figure 3A). Moreover, treatment with (−)-dendroparishiol alone had no effects on NO production in BV-2 cells (Figure 3A). Additionally, treatment with (−)-dendroparishiol showed no cytotoxicity on LPS-induced BV-2 cells (Figure 3B), implying that the reduced NO production observed in (−)-dendroparishiol-treated LPS-stimulated BV-2 cells was not associated with a decreased cell population.

The effects of (−)-dendroparishiol on the proinflammatory cytokine expression (TNF-α and IL-6) in LPS-stimulated BV-2 cells were determined using ELISA. The cells induced with LPS showed significantly higher expression of TNF-α and IL-6, reaching levels of 326.8 ± 10.3 and 58.1 ± 7.7 pg/mL, respectively, compared to the control cells (12.22 ± 2.00 and 2.8 ± 0.7 pg/mL, respectively) (Figure 3C,D). Treatment with (−)-dendroparishiol at the highest dose (5 µM) significantly reduced the expression of TNF-α and IL-6 by 22.1% (254.6 ± 18.4 pg/mL) and 87.5% (7.2 ± 4.1 pg/mL), respectively, compared to the LPS-stimulated group. Additionally, treatment with only (−)-dendroparishiol in BV-2 cells did not affect the levels of TNF-α and IL-6 production.

The effects of (−)-dendroparishiol on iNOS and COX-2 expressions in LPS-stimulated BV-2 cells were analyzed using Western blotting. The protein expression of iNOS and COX-2 was significantly increased by approximately 34% and 64%, respectively, in LPS-stimulated BV-2 cells compared to control cells. Treatment of cells with (−)-dendroparishiol reduced LPS-induced iNOS and COX-2 expressions in a concentration-dependent manner (Figure 3E,F). When compared to the LPS-treated group, treatment with (−)-dendroparishiol at 2.5–5 µM significantly down-regulated the expression of iNOS by 26% and 33% and COX-2 expression by 32% and 51%, respectively. Apart from that, treatment with only (−)-dendroparishiol had no effects on the levels of iNOS and COX-2 in BV-2 cells.

Neuroinflammation is one of the major hallmarks of bacterial meningitis, where microglial cells play a vital role [23]. Microglia are characterized by two distinct polarization profiles: (1) pro-inflammatory (M1) and (2) immunoregulatory (M2). The pro-inflammatory profile involves the expression of cytokines, chemokines, and metabolites, contributing to neurological symptoms [24]. During bacterial meningitis, microglia cells are activated into the M1 form, leading to a cascade of inflammatory mediator release. These mediators are directly involved in the neuronal damage associated with bacterial meningitis [25]. Moreover, LPS is a predominant component of gram-negative bacterial cell walls, such as *Neisseria meningitidis* and *Haemophilus influenza*, causing bacterial meningitis [23]. It stimulates the production of pro-inflammatory cytokines, chemokines, prostaglandins, and NO by microglial cells [26]. Hence, in the present study, LPS-induced BV-2 microglia cells were used as a model of neuroinflammation. In line with the pathophysiology of bacterial meningitis, LPS treatment increased NO, COX-2, and cytokine expression in microglial cells. Treatment with (−)-dendroparishiol significantly reduced the expression of all pro-inflammatory mediators, indicating its potential to attenuate neuroinflammation. Moreover, similar findings were obtained from computer-based predictions of (−)-dendroparishiol’s biological activity. Further, a network pharmacological analysis was performed to provide a holistic overview of the biological pathways involved in the role of (−)-dendroparishiol in bacterial meningitis.

### 2.4. Molecular Docking to Determine the Interaction of (−)-Dendroparishiol with Proinflammatory Enzymes

**Figure 4:**
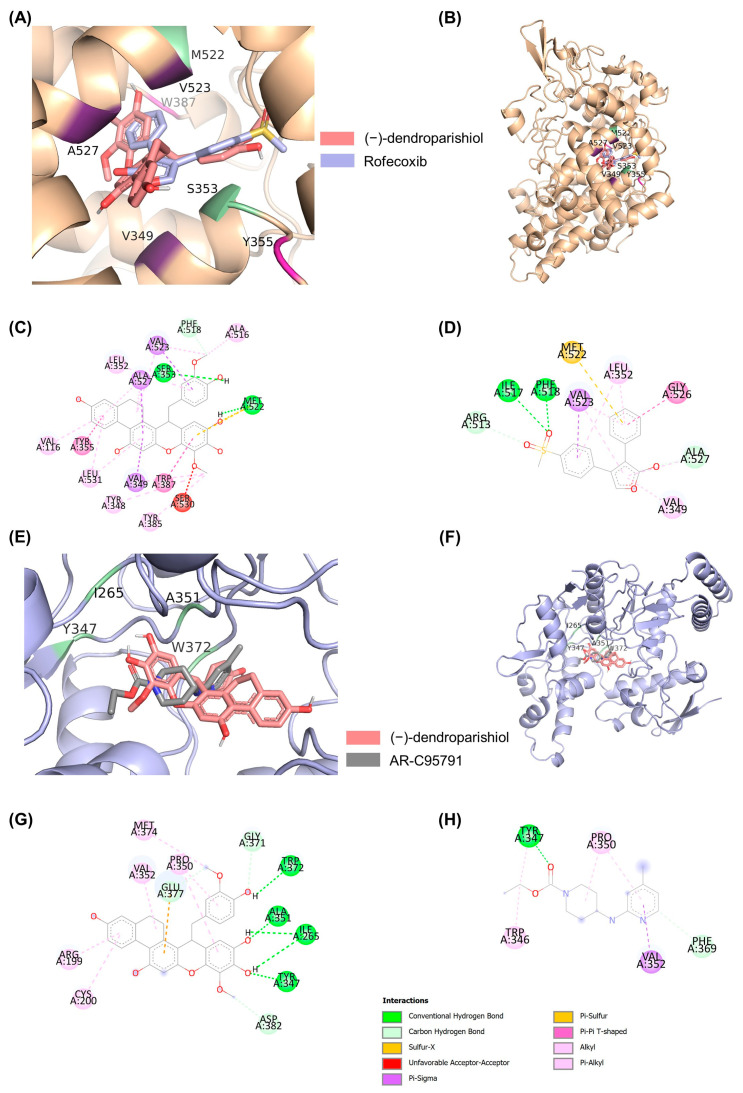
Molecular docking of (−)-dendroparishiol and reference compounds on COX-2 and iNOS. (**A**,**B**) Overlay binding mode of (−)-dendroparishiol and rofecoxib on COX-2. (**C**,**D**) Two-dimensional binding mode showing the interaction of (−)-dendroparishiol (**C**) and rofecoxib with COX-2. (**E**,**F**) Overlay binding mode of (−)-dendroparishiol and AR-C95791 on iNOS. (**G**,**H**) Two-dimensional binding mode showing the interaction of (−)-dendroparishiol (**G**) and AR-C95791 (**H**) with iNOS.

Molecular docking was used to assess the binding of (−)-dendroparishiol to inflammatory enzymes, such as NOS2 (iNOS) and cyclooxygenase 2 (COX-2), the common targets for reducing NO and PGE-2 production, respectively. Table 2 shows the binding profiles of (−)-dendroparishiol to iNOS and COX-2. The results showed comparable binding profiles between (−)-dendroparishiol and reference compounds: iNOS and COX-2 inhibitors. (−)-dendroparishiol and rofecoxib demonstrated a free energy of binding (ΔG) of −11.24 and −10.70 kcal/mol, respectively, to COX-2. The affinity of (−)-dendroparishiol for iNOS (−7.09 kcal/mol) was comparable to that of AR-C95791 (−6.62 kcal/mol). In addition, the binding modes of (−)-dendroparishiol and rofecoxib to COX-2 and (−)-dendroparishiol and AR-C95791 to iNOS showed similarity in location, orientation, and interacting residues (Figure 4 and Table 2). Overall, docking results demonstrate the potential of (−)-dendroparishiol to bind with COX-2 and iNOS and exert anti-inflammatory effects similar to those of selective COX-2 and iNOS inhibitors. Thus, these results indicate that the reduced expression of NO observed in our in vitro experiments could also be due to the ability of (−)-dendroparishiol to reduce the activity of iNOS. This finding is also in agreement with our biological activity prediction data (Table 1) which demonstrated the potential of (−)-dendroparishiol to inhibit iNOS and COX-2 activities.

### 2.5. Network Pharmacology Analysis of the Role of (−)-Dendroparishiol in Bacterial Meningitis

**Figure 5:**
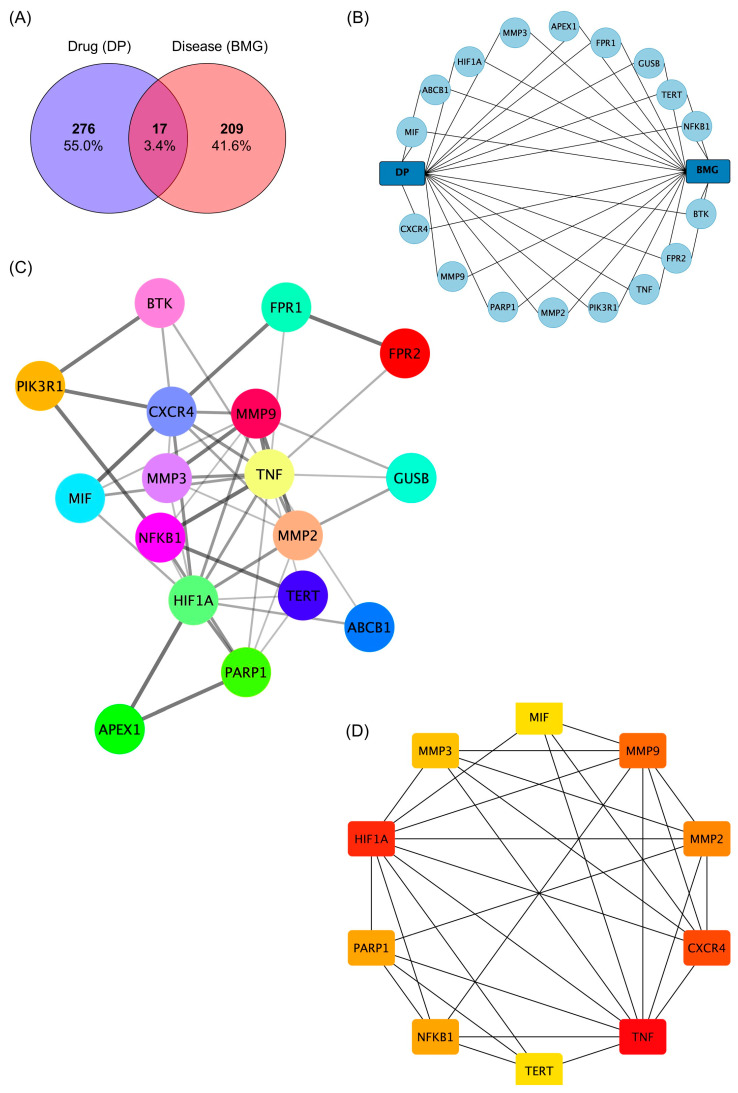
Network analysis of (−)-dendroparishiol target genes and bacterial meningitis differentially expressed genes. (**A**) Venn diagram illustrating (−)-dendroparishiol target genes and bacterial meningitis differentially expressed genes. (**B**) A network showing the interaction between (−)-dendroparishiol, bacterial meningitis, and all potential targets. (**C**) Protein–protein interactions (PPI) network constructed using the STRING database to analyze the interactions between 17 overlapping targets. (**D**) The cytoHubba plug-in generated the top 10 core targets with the highest degree scores. Red to yellow colors indicate a higher or lower degree score. DP, (−)-dendroparishiol; BMG, bacterial meningitis.

The chemical structure of (−)-dendroparishiol was drawn in ChemDraw and further converted into SMILES format. Based on the structure, three target-fishing databases, namely SwissTargetPrediction, SEA SearchServer, and SuperTarget 3.0 were used to predict potential target genes for (−)-dendroparishiol. These three databases are known to have higher recall and precision, indicating higher accuracy in target prediction [27,28]. Ji et al. reported the recall and precision of SwissTargetPrediction and SEA SearchServer at 0.69 and 0.61, and 0.73 and 0.26, respectively, when considering the top five target genes [27]. In addition, the accuracy of the SuperTarget 3.0 database was reported at 80.5% [28]. A total of 293 potential target genes of (−)-dendroparishiol were identified. Furthermore, 226 targets related to bacterial meningitis were obtained from the GeneCard and DisGeNET databases. An analysis of the intercepted targets showed 17 potential targets for (−)-dendroparishiol and bacterial meningitis. Results are represented by a Venn diagram (Figure 5A). A (−)-dendroparishiol-target genes–bacterial meningitis network was constructed concurrently (Figure 5B). The potential role of (−)-dendroparishiol in bacterial meningitis could be via stimulating or inhibiting these target genes.

A protein–protein interaction (PPI) analysis was performed on the STRING database to identify the relationships between the integrated 17 target genes. A total of 46 interactions were identified and depicted as edges (Figure 5C). The average node degree and average node clustering coefficients were calculated as 5.41 and 0.72, respectively, indicating the average number of interactions of proteins in the network and the wellness of the nodes connected to the network. The hub genes were analyzed, and the top 10 genes were selected as core targets using CytoHubba maximal clique centrality (MCC) analysis (Figure 5D). The top 10 genes involved with the activity of (−)-dendroparishiol include tumor necrosis factor (TNF), hypoxia-inducible factor 1-alpha (HF1A), C-X-C chemokine receptor type 4 (CXCR4), matrix metalloproteinase-9 (MMP9), matrix metalloproteinase-2 (MMP2), poly [ADP-ribose] polymerase 1 (PARP1), nuclear factor NF-kappa-B p105 subunit (NFKB1), matrix metalloproteinase-3 (MMP3), telomerase reverse transcriptase (TERT) and macrophage migration inhibitory factor (MIF).

**Figure 6:**
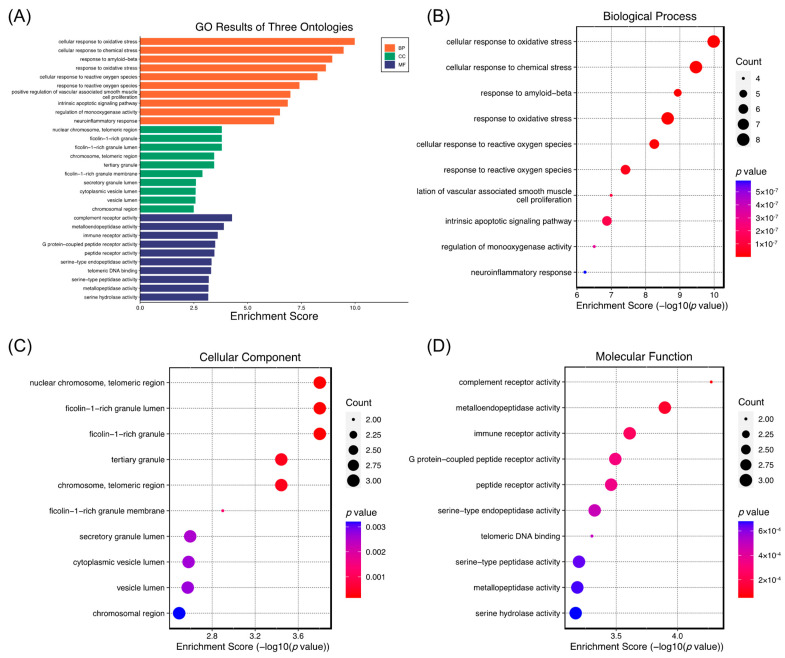
(−)-Dendroparishiol target gene ontology (GO) enrichment analysis. A plot of three enriched ontologies (**A**). Illustrations of enriched biological processes (**B**), cellular components (**C**), and molecular functions (**D**). In each GO term, the circle size represents the number of genes enriched, and the color represents the *p*-value.

Furthermore, GO and KEGG pathway enrichment analyses were performed to obtain a comprehensive understanding of the pathways mediated by (−)-dendroparishiol in bacterial meningitis. A total of 994 biological processes, 36 cellular components, and 90 molecular functions were enriched in GO terms. The top 10 GO terms are summarized in Figure 6. The false discovery rate (FDR)-adjusted *p*-value of all the top ten terms in pathway enrichment analysis were lower than 0.05 (Supplementary Tables S1 and S2), indicating that less than 5% of the significant terms are likely to be false positives. Biological processes such as cellular response to oxidative stress, reactive oxygen species, and neuroinflammatory response were enriched, suggesting their involvement in the biological activity of (−)-dendroparishiol in bacterial meningitis. Neuroinflammation and increased oxidative stress in the brain are the main features of bacterial meningitis, causing neuronal damage and cellular death [2,29]. In oxidative stress conditions, reactive oxygen and nitrite species are increased as a response to the presence of bacteria, leading to cell injury and cellular damage [29]. In addition, inflammation in the brain, manifested by the massive production of proinflammatory mediators, also contributes to the progression of the disease [2]. Several preclinical studies have identified oxidative stress and neuroinflammation as possible targets for improving the progression of bacterial meningitis. According to GO biological process enrichment, (−)-dendroparishiol plays a role in bacterial meningitis primarily through regulating oxidative stress and neuroinflammatory responses. These findings are in line with our in vitro findings and a previous study, which found that (−)-dendroparishiol suppresses oxidative stress in activated macrophages [15].

**Figure 7:**
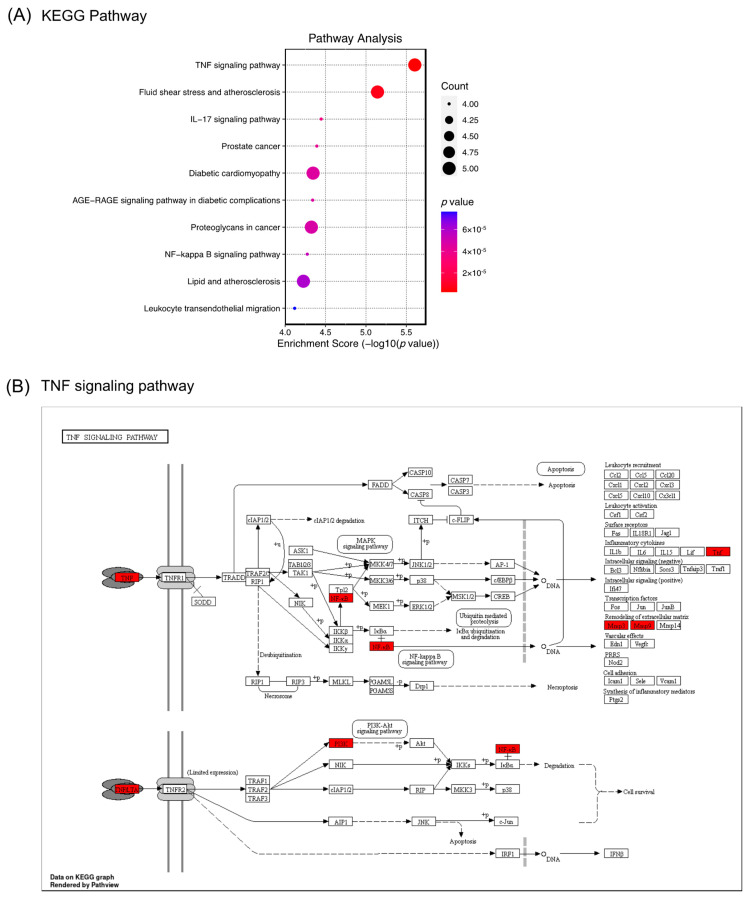
KEGG enrichment analysis of the (−)-dendroparishiol target genes. KEGG pathway enrichment score dot plot (**A**). The size of the circle represents the number of genes enriched in each KEGG pathway, and the color denotes significance. The distribution of genes in the TNF signaling pathway (**B**). ⟶ represents the activation effect, T arrows represent the inhibition effect, and dashed lines represent activation or inhibition effects. The intersection genes are highlighted in red.

Bacterial meningitis is a life-threatening disease with symptoms of sensory-motor deficits and memory impairment. Pharmacological approaches for bacterial meningitis aim not only to kill the bacteria but also to suppress the massive releases of inflammatory mediators [29]. Suppressing inflammation by using dexamethasone, an immunosuppressant, has been reported to improve the condition of patients with acute bacterial meningitis [8]. Since the levels of proinflammatory cytokines, including TNF-α, are increased in bacterial meningitis [30], inhibiting TNF-α and its pathway is beneficial for treating bacterial meningitis. In a preclinical study, a TNF-α converting enzyme inhibitor has been reported to improve neuronal damage and disease symptoms [31]. Therefore, the TNF-α pathway is essential to explore as a potential target, and finding compounds that suppress this pathway is beneficial for treating bacterial meningitis. In the present study, the KEGG pathway analysis indicates 83 enriched KEGG pathways, and the top 10 pathways are shown in Figure 7A. The most prominent pathway of (−)-dendroparishiol in bacterial meningitis is determined to be involved in regulating the TNF signaling pathway (Figure 7B). This result is also in line with our wet lab findings, which showed the ability of (−)-dendroparishiol to suppress TNF-α levels in activated immune cells. Altogether, our results indicate the prominent role of (−)-dendroparishiol in modulating the TNF pathway in bacterial meningitis.

The recently issued network pharmacology evaluation guidelines highlight the importance of providing sufficient scientific evidence to validate the results obtained from network pharmacology analysis [32]. Therefore, despite the in vitro anti-neuroinflammatory effects proven in our study, future studies employing specific inhibitors or gene knockdown techniques both in vitro and in vivo are warranted to validate the proposed molecular mechanisms underlying the effects of (−)-dendroparishiol in bacterial meningitis. In conclusion, our findings demonstrate the potential role of (−)-dendroparishiol in bacterial meningitis through the regulation of neuroinflammatory responses. However, future studies in animal models are recommended to provide comprehensive scientific evidence of the pharmacological activity of (−)-dendroparishiol against bacterial meningitis.

## 3. Materials and Methods

### 3.1. Materials and Chemicals

Lipopolysaccharide (LPS), 3-(4,5-dimethylthiazol-2-yl)-2,5-diphenyltetrazolium bromide (MTT), and other chemicals were purchased from Sigma-Aldrich (St. Louis, MO, USA). Dulbecco’s modified Eagle’s medium (DMEM) was obtained from Invitrogen (Grand Island, NY, USA). ELISA kits for pro-inflammatory cytokine determination were purchased from BioLegend (San Diego, CA, USA).

### 3.2. Prediction of Biological Activity of (−)-Dendroparishiol

Estimation of the biological activity of (−)-dendroparishiol was performed using PASS online http://www.way2drug.com/passonline/ (accessed on 6 January 2023) as previously described [18]. The prediction by PASS online is based on structure-activity relationships using a setting and information of 300,000 compounds.

### 3.3. In Vitro Cell-Based Assay

#### 3.3.1. Extraction and Isolation of (−)-Dendroparishiol from *Dendrobium parishii*

(−)-Dendroparishiol, extracted and isolated from *Dendrobium parishii*, was obtained from Prof. Boonchoo Sritularak, Department of Pharmacognosy and Pharmaceutical Botany, Faculty of Pharmaceutical Sciences, Chulalongkorn University, Thailand. Briefly, the dried and powdered whole plant of *Dendrobium parishii* (2.2 kg) was macerated and extracted with MeOH, and then suspended in water after removal of the solvent. This was partitioned with EtOAc and butanol to obtain ethanol, butanol, and water extracts, respectively, after solution evaporation. Finally, the ethanol extract was fractionated by vacuum liquid chromatography (silica gel, EtOAc-hexane, gradient), yielding seven fractions. An EtOAc-hexane gradient was used to elute the seventh fraction using column chromatography (CC) over silica gel, and 22 fractions were obtained. Using Sephadex LH-20 (acetone), fraction 15 was further fractionated and purified using CC (C-18, MeOH-H2O, 1:1) into (−)-dendroparishiol (5 mg), a red amorphous powder [15].

#### 3.3.2. BV-2 Cell Culture

BV-2 microglial cells were obtained from Accegen Biotechnology (Fairfield, NJ, USA). BV-2 cells were cultured in DMEM containing 10% heat-inactivated fetal bovine serum (FBS) with 1% antibiotic-antimycotic agent. Cells were maintained at 37 °C in a humidified 5% CO_2_ incubator.

#### 3.3.3. Evaluation of Cytotoxic Effects of (−)-Dendroparishiol

The MTT assay was conducted to evaluate the non-toxic concentrations of (−)-dendroparishiol. Cells were seeded in 96-well plates (Costar, NY, USA) at a density of 2 × 10^4^ cells/well. After treatment with different concentrations of (−)-dendroparishiol (1.25–20 µM) for 24 h, the media was removed, and the cells were washed with phosphate buffered saline (PBS). Then the cells were incubated with 0.5 mg/mL MTT solution for 3 h at 37 °C. After incubation, the media was discarded, and dimethyl sulfoxide (DMSO) was added to dissolve the formazan crystals. The absorbance was measured using a microplate reader (CLARIOstar^®^, BMG Labtech, Ortenberg, Germany) at 570 nm.

#### 3.3.4. Hoechst 33342/PI Dual Staining Assay

Briefly, the cells were seeded in 48-well plates at a density of 50,000 cells/well for 24 h. The cells were then incubated with Hoechst 33342 and propidium iodide (PI) (Sigma-Aldrich, St. Louis, MO, USA) before observation under a fluorescence microscope (Olympus IX51 inverted microscope, Tokyo, Japan).

#### 3.3.5. Anti-Inflammatory Effects of (−)-Dendroparishiol on LPS-Stimulated BV-2 Cells

The cells were seeded at a density of 2 × 10^5^ cells/well in 24-well plates (Costar, NY, USA) and incubated at 37 °C in a humidified incubator under a 5% CO_2_ atmosphere for 24 h. The cells were treated with (−)-dendroparishiol at concentrations of 1.25, 2.5, and 5 µM for 1 h. LPS at a concentration of 1 µg/mL was added and incubated for 24 h. The culture media was collected to measure nitric oxide (NO) release using the Griess reaction and TNF-α and IL-6 expression by enzyme-linked immunosorbent assay (ELISA).

#### 3.3.6. Nitrite Concentration Analysis Using Griess Reagent

The nitrite concentration in the culture medium was analyzed as an indicator of nitric oxide levels using the Griess reaction. Briefly, 50 µL of sulfanilamide (1% in 5% phosphoric acid) was added to 100 µL of the culture medium in each well of a 96-well plate and incubated for 5 min in the dark. Thereafter, 50 µL of N-1-Napthylenediamine dihydrochloride (NED) solution was added, followed by incubation for 5 min in the dark. The absorbance was measured at 520 nm. The NO concentration in the culture media was determined by using a standard curve for NaNO_2_.

#### 3.3.7. Determination of IL-6 and TNF-α Levels Using ELISA

The levels of TNF-α and IL-6 were determined in the culture medium using ELISA kits (BioLegend) as directed by the manufacturer. The cytokine concentration was determined by using their respective standard curves. The absorbance was measured using a microplate reader at 450 nm.

#### 3.3.8. Western Blot Analysis

For Western blot analysis, the cells were seeded at a density of 1 × 10^6^ cells/well in 6-well plates. After the treatments, the cell culture media was removed, and the cells were lysed by adding lysis buffer (60 μL). The plates were incubated at 4 °C for 30 min with shaking. The cell lysates were collected using scrapers, centrifuged (4 °C, 13,500× *g*, 10 min), and the supernatants were collected for Western blot analysis. After determining the protein concentration in each sample by applying the BCA assay, 40 μg of proteins were loaded and electrophoresed on 8% sodium dodecyl sulfate-polyacrylamide gels (SDS-PAGE). Proteins were transferred onto nitrocellulose membranes using a wet transfer system, and the membranes were incubated with 5% skimmed milk in TBST (Tris Buffered Saline with 0.1% Tween 20) for 2 h to block the non-specific binding sites. Then the membranes were incubated with primary antibodies (iNOS, COX-2, and β-actin) at 4 °C overnight, followed by incubation with horseradish peroxidase-labeled secondary antibodies for 2 h at room temperature. Finally, the protein bands were visualized on a medical X-ray cassette with Kodak Green 400 Screen (Rochester, NY, USA) using detection reagents. β-actin was used as the loading control for normalizing the signal intensity of the bands, and the relative ratio of band intensity between the marker proteins and β-actin was expressed as shown in the results.

### 3.4. Molecular Docking

In the present study, molecular docking was performed to assess the binding of (−)-dendroparishiol to two protein targets, COX-2 and iNOS. The 2D and 3D structures of (−)-dendroparishiol were generated using ChemDraw Professional 17.0 and Chem3D 17.0, respectively. The 3D structure was subjected to energy minimization using Chem3D 17.0 and then exported to a PDB file. The PDB file was converted to a PDBqt file using AutoDock 4.2. The crystal 3D structures of COX-2 and iNOS were obtained from the protein data bank (PDB) with PDB codes 5kir and 3EG7, respectively. The proteins were adjusted by eliminating other small molecules and water molecules, and adding polar hydrogen atoms and Gasteiger charges using AutoDock4.2. The docking method was further validated by the redocking approach. (−)-Dendroparishiol and reference compounds (rofecoxib for COX-2 and AR-C95791 for iNOS) were docked with the prepared proteins using AutoDock 4.2. The parameters used in the molecular docking were: number of genetic algorithm (GA) runs: 100, population size: 300, the maximum number of generations: 27,000, the maximum number of energy evaluations per run: 2,500,000 (COX-2) and 25,000,000 (iNOS), and reference root-mean-square deviation (RMSD) tolerance: 2.0 Å. The best pose with the lowest binding energy was selected for further visualization using PyMol.

### 3.5. Network Pharmacology Analysis

#### 3.5.1. Identification of (−)-Dendroparishiol-Target Genes

The target genes of (−)-dendroparishiol were identified using several web-based target prediction tools, including SwissTargetPrediction (http://www.swisstargetprediction.ch/, accessed on 5 January 2023), SEA SearchServer (https://sea.bkslab.org/, accessed on 5 January 2023) [33], and SuperTarget (https://prediction.charite.de/subpages/target_prediction.php, accessed on 5 January 2023) [34]. A UniProt database search (https://www.uniprot.org/, accessed on 5 January 2023) was performed to obtain gene symbols for potential targets. Duplicate (−)-dendroparishiol target genes were removed before analysis.

#### 3.5.2. Identification of Bacterial Meningitis-Target Genes

It is essential to identify genes that are linked to diseases before developing a compound-target network. Therefore, information related to bacterial meningitis-associated target genes was collected from two databases: (1) GeneCards (https://www.genecards.org/, accessed on 9 January 2023), a human gene database containing information about all annotated and predicted genes in humans (score ≥ 10), and (2) DisGeNET database (https://www.disgenet.org/, accessed on 9 January 2023) (fit score ≥ 0.1). The UniProt database was used to further harmonize and standardize target genes obtained from all databases. The genes obtained from all databases were compiled, and duplicate bacterial meningitis target genes were removed.

#### 3.5.3. Construction of Protein–Protein Interaction (PPI) Network

Initially, an online Venn diagram tool (https://bioinfogp.cnb.csic.es/tools/venny/, accessed on 9 January 2023) was used to visualize and identify the interaction between bacterial-meningitis-related targets and (−)-dendroparishiol targets. Based on the interaction data, an interactive network of (−)-dendroparishiol targets and bacterial-meningitis-related targets was constructed, and the network was visualized using Cytoscape (version 3.9.1). The top 10 genes with a high level of interaction were determined using Cytoscape’s cytoHubba plugin v.0.1 [35].

#### 3.5.4. GO Enrichment and Pathway Analysis

Kyoto Encyclopedia of Genes and Genomes (KEGG) and Gene Ontology (GO) pathway enrichment analyses were used to illustrate possible mechanisms of action and pathways involved in the anti-meningitis activity of (−)-dendroparishiol. The KEGG pathway enrichment analysis was conducted as described previously to determine the possible molecular mechanism of (−)-dendroparishiol in bacterial meningitis [36]. Through GO analysis, genes were examined for their functional roles in biological processes, cellular components, and molecular functions. An online tool for bioinformatics data analysis (http://www.bioinformatics.com.cn/, accessed on 10 January 2023) was used to perform GO and KEGG pathway enrichment analyses.

### 3.6. Statistical Analysis

All cell culture results are shown as representative of three independent experiments and indicated as mean ± SD values. These results were analyzed by one-way ANOVA and post hoc test, respectively. Statistical analysis was performed using GraphPad Prism, and the statistical significance was considered to be achieved when *p* < 0.05. (95% of confidence level).

## Figures and Tables

**Table 1:** The predicted biological activities of (−)-dendroparishiol associated with inflammatory pathway.

Pa	Pi	Activity
0.806	0.008	JAK2 expression inhibitor
0.620	0.004	NOS2 expression inhibitor
0.437	0.041	MMP9 expression inhibitor
0.335	0.010	Nitric oxide antagonist
0.405	0.093	Anti-inflammatory
0.333	0.083	TNF expression inhibitor
0.285	0.048	Non-steroidal anti-inflammatory agent
0.213	0.024	Transcription factor NF kappa B inhibitor
0.187	0.020	Lipoxygenase inhibitor
0.172	0.014	Cytokine release inhibitor
0.175	0.040	Leukotriene synthesis inhibitor
0.145	0.055	Interleukin 4 antagonist
0.107	0.023	5-Lipoxygenase inhibitor
0.210	0.129	Phospholipase C inhibitor
0.158	0.095	Nitric oxide scavenger
0.111	0.076	Cyclooxygenase inhibitor
0.076	0.072	Cyclooxygenase 2 inhibitor

**Table 2:** The binding profiles of (−)-dendroparishiol with COX-2 and iNOS.

Protein Targets	Ligands	∆G (kcal/mol)	Interacting Residues							
			H-Bond	C-H Bond	Pi-Sulfur	Pi-Anion	Pi-Pi	Amide-Pi	Pi-Sigma	Hydrophobic
COX-2	(−)-dendroparishiol	−11.24	SER353 MET522	PHE518	MET522	-	TYR355 TRP387	-	VAL349 VAL523 ALA527	VAL116 TYR348 LEU352 TYR385 ALA516 LEU531
	Rofecoxib 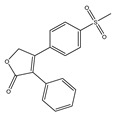	−10.70	ILE517 PHE518	ARG513 ALA527	MET522	-	-	GLY526	VAL523	VAL349 LEU352
iNOS	(−)-dendroparishiol	−7.09	ILE265 TYR347 ALA351 TRP372	GLY371 GLU377 ASP382	-	GLU377	-	-	-	ARG199 CYS200 PRO350 VAL352 MET374
	AR-C95791 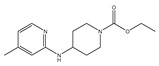	−6.62	TYR347	PHE369	-	-	-	-	VAL352	TRP346 PRO350

## Data Availability

Not applicable.

## Supplementary Materials

The following supporting information can be downloaded at: https://www.mdpi.com/article/10.3390/ijms24098072/s1.

## Abbreviations

DP	*Dendrobium parishii*
NF-κB	Nuclear factor kappa
IL-1β	Interleukin-1beta
IL-6	Interleukin-6
IL-1	Interleukin-1
TNF-α	Tumor necrosis factor alpha
NO	Nitric oxide
iNOS	Inducible nitric oxide synthase
COX-2	Cyclooxygenase-2
LPS	Lipopolysaccharide
SOD	Superoxide dismutase
GPx	Glutathione peroxidase
CAT	Catalase
MTT	3-(4,5-Dimethylthiazol-2-yl)-2,5-diphenyltetrazolium bromide
MMPs	Matrix metalloproteinases
ROS	Reactive oxygen species
JAK2	Janus kinase 2
PGE-2	Prostaglandin E2
BMG	Bacterial meningitis
DMSO	Dimethyl sulfoxide
ELISA	Enzyme-linked immunosorbent assay
PPI	Protein–protein interactions
MCC	Maximal clique centrality
GO	Gene Ontology
KEGG	Kyoto Encyclopedia of Genes and Genomes
FDR	False discovery rate
SDS-PAGE	Sodium dodecyl sulfate-polyacrylamide gels
TBST	Tris Buffered Saline with 0.1% Tween 20
PDB	Protein data bank
DMEM	Dulbecco’s modified Eagle’s medium
FBS	Fetal bovine serum
PBS	Phosphate buffered saline
PI	Propidium iodide
NED	N-1-Napthylenediamine dihydrochloride
BCA	Bicinchoninic acid
