# Supplementary material for: An Integrative Approach to Investigate the Mode of Action of (−)-Dendroparishiol in Bacterial Meningitis: Computer-Aided Estimation of Biological Activity and Network Pharmacology
Source: Int J Mol Sci. 2023 Apr 29;24(9):8072. doi: 10.3390/ijms24098072 (PMC10179348; doi:10.3390/ijms24098072)
Supplement: Supplementary file 1 [file ijms-24-08072-s001.zip › ijms-2274754-supplementary.pdf]

Supplementary Information for

An integrative approach to investigate the mode of action of (−)-dendroparishiol in bacterial meningitis: computer-aided estimation of biological activity and network pharmacology

Table S1: Summary of top ten GO terms associated with the biological activity of (−)-dendroparishiol in bacterial meningitis.

Category	Pathway ID	Pathway Description	p.adjusted	FDR
GO_BP	GO:0034599	Cellular response to oxidative stress	1.708025E-07	7.11518E-08
	GO:0062197	Cellular response to chemical stress	2.80014E-07	1.16646E-07
	GO:1904645	Response to amyloid-beta	6.33716E-07	2.63989E-07
	GO:0006979	Response to oxidative stress	9.36702E-07	3.90205E-07
	GO:0034614	Cellular response to reactive oxygen species	1.8377E-06	7.65539E-07
	GO:0000302	Response to reactive oxygen species	1.06021E-05	4.41656E-06
	GO:1904707	Positive regulation of vascular associated Smooth muscle cell proliferation	2.37983E-05	9.91373E-06
	GO:0097193	Intrinsic apoptotic signaling pathway	2.76511E-05	1.15187E-05
	GO:0032768	Regulation of monooxygenase activity	5.74165E-05	2.39182E-05
	GO:0150076	Neuroinflammatory response	9.64407E-05	4.01746E-05
GO_CC	GO:0000784	Nuclear chromosome, telomeric region	0.003698013	0.001890713
	GO:0101002	Ficolin-1-rich granule	0.003698013	0.001890713
	GO:1904813	Ficolin-1-rich granule lumen	0.003698013	0.001890713
	GO:0000781	Chromosome, telomeric region	0.0050541	0.002584051
	GO:0070820	Tertiary granule	0.0050541	0.002584051
	GO:0101003	Ficolin-1-rich granule membrane	0.014729917	0.007531085
	GO:0034774	Secretory granule lumen	0.020757477	0.010612845
	GO:0060205	Cytoplasmic vesicle lumen	0.020757477	0.010612845
	GO:0031983	Vesicle lumen	0.020757477	0.010612845
	GO:0098687	Chromosomal region	0.022444652	0.011475461
GO_MF	GO:0004875	Complement receptor activity	0.008164383	0.003571569
	GO:0004222	Metalloendopeptidase activity	0.009772931	0.00427524
	GO:0140375	Immune receptor activity	0.010436213	0.004565397
	GO:0008528	G protein-coupled peptide receptor activity	0.010436213	0.004565397
	GO:0001653	Peptide receptor activity	0.010436213	0.004565397
	GO:0004252	Serine-type endopeptidase activity	0.010436213	0.004565397
	GO:0042162	Telomeric DNA binding	0.010436213	0.004565397
	GO:0008236	Serine-type peptidase activity	0.010436213	0.004565397
	GO:0008237	Metallopeptidase activity	0.010436213	0.004565397
	GO:0017171	Serine hydrolase activity	0.010436213	0.004565397

Table S2. Summary of top ten significant KEGG pathway terms modulated by (−)-dendroparishiol in bacterial meningitis

Pathway ID	Pathway Description	p.adjusted	FDR
hsa04668	TNF signaling pathway	0.000404638	0.000209048
hsa05418	Fluid shear stress and atherosclerosis	0.000586342	0.000302921
hsa04657	IL-17 signaling pathway	0.001076687	0.000556248
hsa05215	Prostate cancer	0.001076687	0.000556248
hsa05415	Diabetic cardiomyopathy	0.001076687	0.000556248
hsa04933	AGE-RAGE signaling pathway in diabetic complications	0.001076687	0.000556248
hsa05205	Proteoglycans in cancer	0.001076687	0.000556248
hsa04064	NF-kappa B signaling pathway	0.001076687	0.000556248
hsa05417	Lipid and atherosclerosis	0.001076687	0.000556248
hsa04670	Leukocyte transendothelial migration	0.001244516	0.000642953
